# Follicular adenoma in a thyroglossal duct

**DOI:** 10.1016/S1808-8694(15)30091-4

**Published:** 2015-10-19

**Authors:** Rogério Aparecido Dedivitis, Fábio Rocha Lima, Cristiano Rosa Guirado

**Affiliations:** a PhD in Medicine - Otolaryngology and Head and Neck Surgery Graduate Course - UNIFESP – Paulista School of Medicine. MD; b Pathologist graduated from the University of São Paulo. Assistant Physician – Pathology Department of the Ana Costa Hospital; c Resident physician in Otorhinolaryngology - Centro Médico Aquino - CEMA, São Paulo. Head and Neck Surgery Department

**Keywords:** follicular adenoma, surgery, thyroglossal duct, thyroid neoplasms

## INTRODUCTION

The thyroglossal Duct (TD) cyst results from a failure in obliterating the embryogenic duct produced during thyroid migration. A cyst may develop from the secretory residual epithelium. Ectopic thyroid tissue neoplasias are rare, and even rarer when associated with the TD. It has been reported that over 7% of adults have some remains of the TD1 and over 62% of them may have some ectopic thyroid tissue^2^. Most neoplasias that happen on the TD are made up of papillary carcinomas, responding for 91% of the cases reported^2^. Here, we report on a case of a follicular adenoma involving a TD.

## CASE REPORT

An 18 year old patient noticed a painless mass in the mid-line of his neck for about one month. He had no prior history of neck injury. He did not complain of dysphagia or odynophagia, or of phlogistic signs. During the clinical exam, we noticed a deep nodular lesion, of about 3x2cm, fibroelastic, mobile during swallowing and at tongue protrusion, in the mid-line, at the height of the thyrohyoid membrane. There were no clinically significant nodes. The thyroid gland was normal at palpation. Ultrasound showed a cystic mass, with a thick content; and the thyroid was within normal limits.

During surgery, the TD was identified and resected by means of the Sistrunk maneuver, including the removal of the central hyoid bone and the entire cyst tract, all the way to the foramen cecum, at the base of the tongue. Intraoperative frozen section histopathology showed a solid neoplastic lesion of follicular pattern.

**Figure 1:**
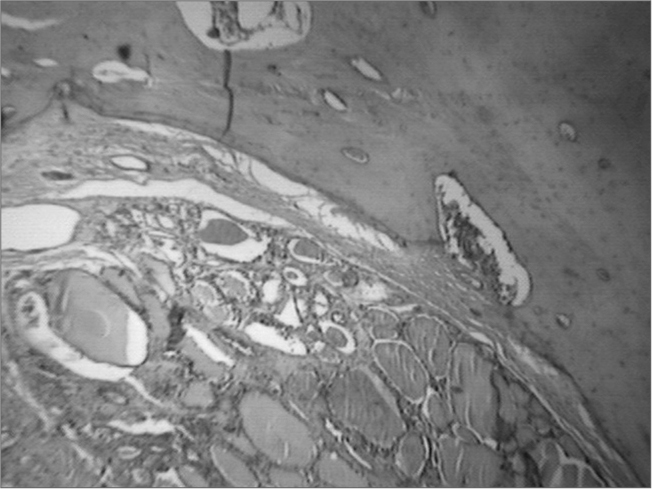
Histopathology showed enlarged follicles (F), full of a colloid substance outlined by atrophic columnar epithelium, matching the description of a follicular adenoma, without signs of atypia and capsular invasion. The mass is attached to a typical trabecular bone - hyoid bone (H). HE, 100x.

Histopathology showed enlarged thyroid follicles, full of colloid and outlined by atrophic columnar epithelium, matching a diagnosis of follicular adenoma. The mass continued a trabecular-type of bone corresponding to the hyoid bone body. We did not notice atypia or capsular invasion (Figure 1). The patient was discharged from the hospital on the first day of postoperative in sound health and remained asymptomatic for 30 months

## DISCUSSION

The thyroid gland appears on the third week of embryologic development, on the floor of the primitive pharynx, it descends as a bilobed diverticulum in order to remain ventral to the trachea, around the seventh week. The gland remains attached to the foramen cecum by the TD from the eighth to the tenth week, when it usually recedes. The TD cyst results from a failure in this involution. TD-associated neoplasia is rare and make up less than 1% of the thyroid cancers^3^.

Since the TD cyst is closely related to the thyroid gland development, thyroid tissue may be found on its wall. Although it is a plausible cause for cancer development in the ectopic thyroid tissue on the cyst wall, this fact is yet to be determined. It may be postulated that TD remains, having an embryogenic potential for thyroid genesis, may produce a neoplasia that is typically of thyroid origin^4^.

The physical exam usually reveals a palpable and firm mass, of slow and progressive growth, located on the mid-line, near the hyoid bone; it is usually mobile at tongue protrusion and swallowing. TD cyst carcinoma is not usually suspected in the clinical practice and its diagnosis is usually done after removal of the surgical specimen. A cyst accelerated growth rate may call the physician’s attention to TD malignancy. Current consensus considers the TD as a recurrent growth source for carcinomas, instead of being the extension of a thyroid tumor^5^.

The ultrasound exam may provide accurate information without the need for other radiologic procedures that take time and are expensive. TD cyst is not a simple cyst. Its aspect may vary from a typically anechoic image, all the way to a pseudocyst image, as seen in our case. The presence of a solid or mixed structure is frequently described in TD benign cyst under an inflammatory process. The Sistrunk procedure for thyroglossal tract resection up to the foramen cecum, on the tongue base, including the hyoid bone central portion, is well accepted because of its good results, with low recurrence rates. Frozen-section intraoperative histopathology is useful in assessing malignancy^6^.
